# Comparison between rapid and mixed maxillary expansion through an assessment of arch changes on dental casts

**DOI:** 10.1186/s40510-015-0089-6

**Published:** 2015-06-27

**Authors:** Vincenzo Grassia, Fabrizia d’Apuzzo, Abdolreza Jamilian, Felice Femiano, Lorenzo Favero, Letizia Perillo

**Affiliations:** Multidisciplinary Department of Medical-Surgical and Dental Specialties, Second University of Naples, Via Luigi De Crecchio 6, 80138 Napoli, Italy; Department of Orthodontics, Dental Branch, Craniomaxillofacial Center Islamic Azad University, No.2713, Vali Asr St, Tehran, Iran; Department of Neurosciences, University of Padua, Via Giustiniani 2, 35128 Padova, Italy; Multidisciplinary Department of medical-Surgery and Dental Specialties, Second University of Naples; Post-Graduate Orthodontics Programme and Operative Unit, Via Luigi De Crecchio 6, 80138 Napoli, Italy

## Abstract

**Background:**

Aim of this retrospective observational study was to compare upper and lower dental changes in patients treated with Rapid Maxillary Expansion (RME) and Mixed Maxillary Expansion (MME), assessed by dental cast analysis.

**Methods:**

Treatment groups consisted of 42 patients: the RME group (*n* = 21) consisted of 13 female and 8 male subjects with the mean age of 8.8 years ± 1.37 at T0 and 9.6 years ± 1.45 at T1; the MME group (*n* = 21) consisted of 12 female and 9 male patients with a mean age of 8.9 years ± 2.34 at T0 and 10.5 years ± 2.08 at T1. The upper and lower arch analysis was performed on four dental bilateral landmarks, on upper and lower casts; also upper and lower arch depths were measured. The groups were compared using independent sample t-test to estimate dental changes in upper and lower arches.

**Results:**

Before expansion treatment (T0), the groups were similar for all examined variables (p>0.05). In both RME and MME group, significant increments in all the variables for maxillary and mandibular arch widths were observed after treatment. No significant differences in maxillary and mandibular arch depths were observed at the end of treatment in both groups. An evaluation of the changes after RME and MME (T1) showed statistically significant differences in mandibular arch depth (p<0.001) and maxillary intercanine widths (p<0.05). Differences in maxillary arch depth and arch width measurements were not significant.

**Conclusions:**

RME and MME can be considered two effective treatment options to improve transverse arch dimensions and gain space in the dental arches. A greater lower arch expansion was observed in the MME group, which might be attributed to the “lip bumper effects” observed in the MME protocol.

## Background

Dento-skeletal maxillary constriction can be treated by opening the mid-palatal suture, and widening the roof of the mouth and floor of the nose [1–5].

Skeletal expansion allows the correction of a posterior crossbite, when present [6–10]. In addition, a space gain is achieved in the arch, and the underlying permanent tooth buds are buccally repositioned [11–14].

Maxillary expansion can be performed with different methods: rapid maxillary expansion (RME) [15–17], semirapid maxillary expansion (SRME) [18], slow maxillary expansion (SME) [19–21] and mixed maxillary expansion (MME) [22–24]. Each treatment protocol is based on a different rationale, but all produce both skeletal and dental changes.

MME separates the two maxillary halves at the first appointment, by applying all the expansion forces to maxillary bones, thereby increasing skeletal effects and reducing unwanted dental side movements. In a previous study comparing MME-induced dento-skeletal changes on postero–anterior cephalograms in pre-pubertal patients with those in an untreated control group [22], MME was found to be an effective treatment option for improving dento-skeletal transverse dimensions and correcting posterior crossbite. These positive outcomes were associated with major skeletal and minor dental changes. A further research comparing the arch changes on dental casts, using the same sample and treatment procedures, confirmed the validity of MME as a useful alternative protocol for improving transverse arch dimensions and correcting posterior crossbite, as well as for gaining arch space to relieve mild-to-moderate tooth-size/arch discrepancies [23]. A third paper compared the dento-skeletal changes on postero–anterior cephalograms in two groups of pre-pubertal patients treated with MME or RME [24, 25]. Both expansion procedures proved effective in increasing skeletal transverse dimensions by opening the mid-palatal suture in growing patients. However, MME had significantly fewer dental side effects than RME. To complete this latter research, in the light that one of the outcomes of expansion is often to increase arch widths and achieve space gain [26–31], it could be useful to investigate whether differences in arch changes were present.

Thus, the aim of this study was therefore to compare the changes in arch widths on dental casts in two groups of pre-pubertal patients treated with MME or RME.

## Methods

Appropriate ethical approval was secured by the Health Research Ethics Board of the Second University of Naples (No.429), in July 22, 2014.

Consecutive records of patients treated with maxillary expansion at the Orthodontic Unit of the Second University of Naples, Italy, and in a private practice (LP) from October 2010 to March 2012 were examined. The parents of all children involved in the study gave informed consent. Inclusion criteria of this retrospective study were unilateral or bilateral posterior crossbite and/or variable degree of crowding, mixed dentition with fully erupted upper permanent molars, Cervical Vertebral Maturation Stage (CVMS) 1 or 2, treatment performed with RME or MME, dental casts, high quality latero–lateral, postero–anterior and occlusal radiographs at pre-expansion (T0) and post-expansion (T1). Exclusion criteria were cranio-facial anomalies or syndromes, periodontal diseases, dental trauma or anomalies and previous orthodontic treatment.

**Fig. 1:**
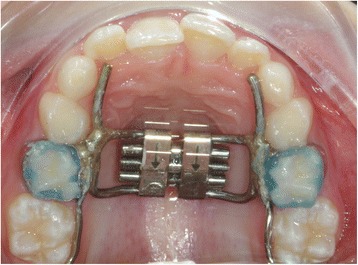
Palatal Hyrax-type expander

Forty-two patients met the inclusion criteria. Of these, 21 were treated with RME and 21 with MME using a palatal Hyrax-type expander (Fig. 1) applied in mixed dentition and bonded to the first upper molars and either first deciduous molars or first bicuspids.

### Activation

In both groups, activation started immediately after the appliance was fitted, and ended when overcorrection was achieved with the palatal cusps of the upper molars riding up on the buccal cusps of the lower molars. The activation schedule of both protocols is reported in Table 1.

**Table 1:** Characteristics of RME and MME sample groups

	Expansion				Retention	Total treatment duration
	Phase	Turns	Duration	Controls		
RME group	One: rapid	2/day	1–3 weeks	4/months	8 months ± 2	1.2 year ± 0.3
MME group	First: very rapid	4–2–1/day	1 h	2/months	8 months ± 2	1.3 year ± 0.2
	Second: slow	2/week	4–6 months			

### RME group activation

In the RME group, the operator began activation at the chairside with two turns of the expansion screw (0.25 mm per turn). Parents were then instructed to continue activation at home with two turns per day.

During the expansion phase, which lasted from 1 to 3 weeks depending on the degree of maxillary constriction and/or crowding, patients were monitored once a week.

### MME group activation

In the MME group, the protocol comprised two phases: an initial, very rapid opening of the device followed by a second, slower activation. The first phase, started at the chair side, included three steps with four, two and one turn (0.25 mm per turn), respectively. The three steps were carried out at the same appointment until the suture was opened.

The second expansion phase then began and parents were instructed to continue activation at home with one turn every 3 days.

During the expansion phase, which lasted from 4 to 6 months depending on the degree of maxillary constriction and/or of crowding, patients were monitored once every 2 weeks.

### Clinical procedure for MME

After the first step, the patient usually experiences tenderness of the bonded teeth, which disappears in 20 to 30 min. After the second step, tenderness lasting 10–15 min then shifts to the palatal incisor area and, finally, following the third step, to the suture area. A decrease in tenderness of the bonded teeth and/or tenderness in the sutural area may indicate that the maxillary halves have been separated. However, a successful separation can only be confirmed by occlusal radiograph, before and after maxillary expansion. In the event of increased palatal suture resistance, a fourth step with two additional turns may be performed. In this case, tenderness in the temporo- and fronto-zygomatic areas may be experienced. The number of steps and associated turns therefore depends on suture intercuspidation.

### Retention phase

After the expansion phase, the Hyrax device was removed in both groups in order to stabilize the screw with acrylic. The device was then re-cemented so that it could be used as a retainer. The retention phase lasted on average 8 months. The retention schedule of both protocols is shown in Table 1.

### Data collection

Digital occlusal photographs were taken of all maxillary and mandibular casts according to a standard technique and imported into Adobe Photoshop, version 5.0 (Adobe Systems, San Jose, CA). Each photograph was saved with a 5-mm grid and then imported into Scion Image (Scion, Frederick, MD; a version of the Macintosh program, NIH Image, from the National Institutes of Health). Each point in Scion was recorded to an x, y coordinate system and imported into a Microsoft Excel program to orient the coordinates and align the maxillary and mandibular casts. The dental casts were measured at T0 and T1 for each patient.

### Landmark identification

**Fig. 2:**
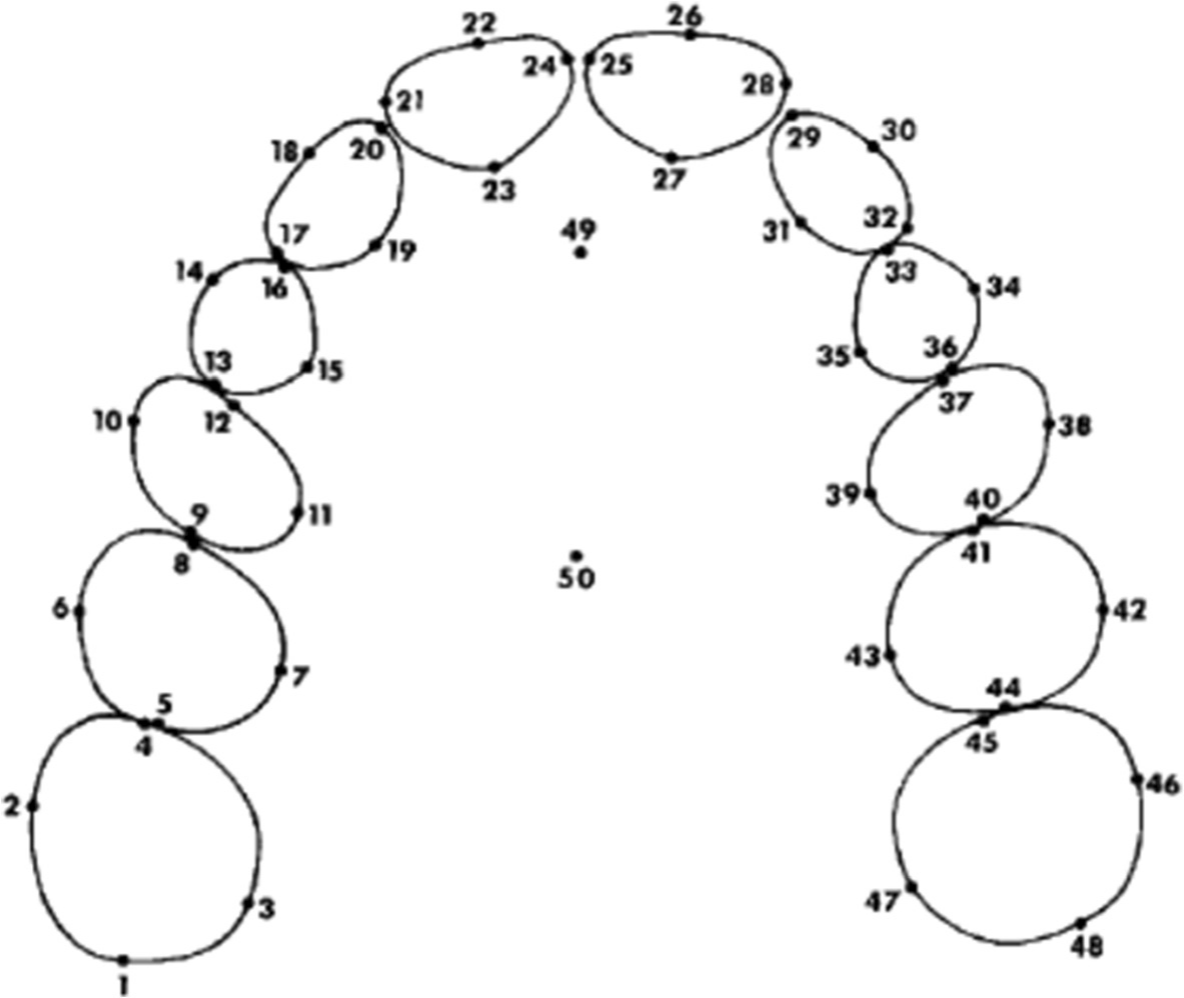
Location of landmarks on maxillary dental casts. Similar dental landmarks were located on the mandibular dental arch

Landmarks were identified on the distal, facial, mesial and lingual surfaces of each tooth from the right first permanent molar to the left first permanent molar in the same arch (Fig. 2). These points were selected in accordance with guidelines reported in literature to determine the geometric centre of each tooth, known as the tooth centroid. This point provides a more accurate measurement of arch width as it removes the effect of tooth rotation.

Landmarks were not recorded if the teeth were in the process of exfoliation, ectopically erupted or in the process of eruption if the height of the four outer surfaces (distal, facial, mesial and lingual) were visible.

### Measurements

**Fig. 3:**
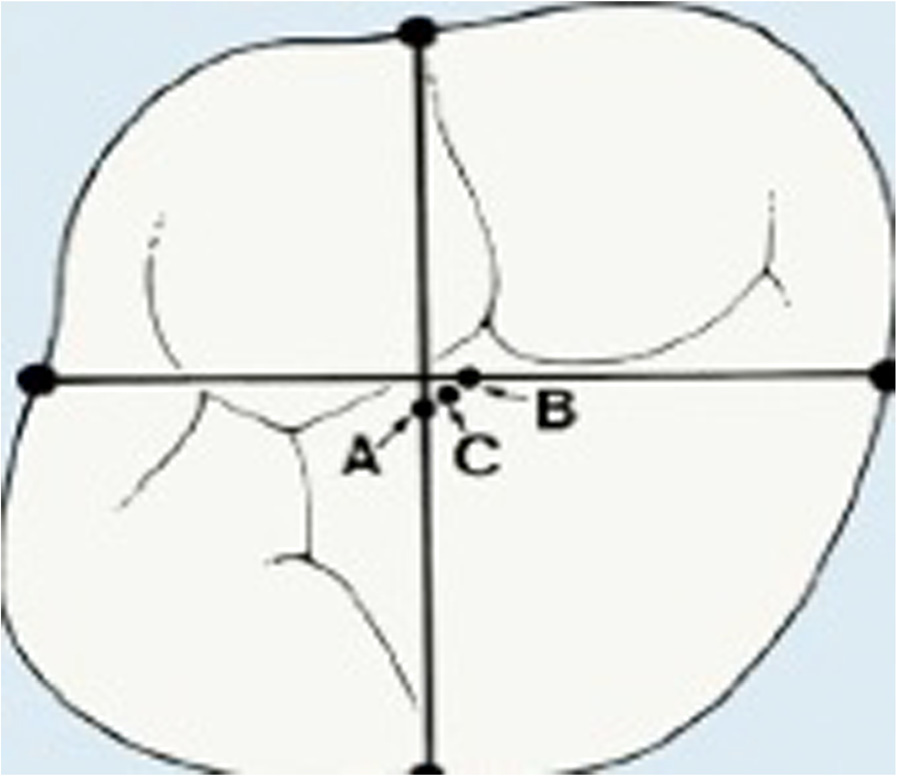
Location of the centroid of each posterior tooth. (A) Midpoint of a line connecting mesial and distal landmarks. (B) Midpoint between buccal and lingual landmarks. (C) Centroid located midway between points A and B

For each dental arch, at T0 and T1, four transversal linear measurements (arch widths) connecting the centroid of a tooth (Fig. 3) and its antimere, and one sagittal measurement (arch depth) were analysed.

For each tooth, the midpoint of a line connecting mesial and distal landmarks was determined, and another midpoint was constructed midway between the buccal and lingual landmarks of the tooth; the centroid is located midway between the midpoints. In the majority of teeth studied, the centroid and the midpoint between the approximal midpoints coincided or were very close to each other.

Arch width was measured between the following teeth on both arches: primary/permanent canines, first primary molars/first premolars, second primary molars/second premolars, and first permanent molars.

**Fig. 4:**
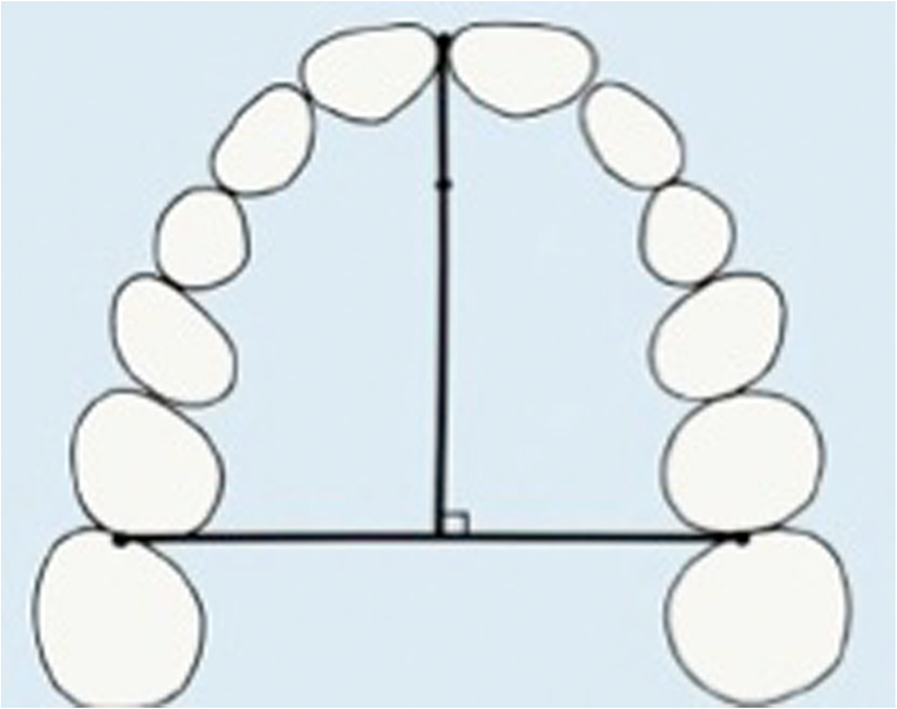
Arch depth measured as distance from a point midwaybetween facial surfaces of central incisors to a line tangent to mesialsurfaces of first permanent molars

Arch depth was determined by measuring the length of a perpendicular line constructed from the contact point between mesial contact points of central incisors to a line connecting points between second premolars and first molars (Fig. 4).

### Error method

All measurements were carried out by the same operator. A combined error of the landmark location and tracing was determined. The method error was calculated from the double determinations of 12 randomly selected dental casts, re-measured after an interval of 1 week, using Dahlberg’s formula. The mean value of the method error was 0.5 ± 0.2 mm, within acceptable limits for the analysis of serial dental casts.

### Statistical analysis

Descriptive statistics were performed on dental cast measurements at T0 and T1 for RME and MME groups.

Statistical comparisons were performed on:

- starting forms: RME group vs. MME group at T0
- treatment effects: RME group T1–T0
- treatment effects: MME group T1–T0
- final forms: RME group vs. MME group at T1.

All data were tested as for the normality; therefore, a parametric test was applied for the comparisons of the groups. Comparison of continuous variables between groups was made through unpaired *t*-tests, while changes within groups were tested through paired *t*-tests. The level of statistical significance set at *p* < .05 for all statistical tests. The statistical Package for the Social Sciences (version 22; SPSS, Chicago, III) was used for data analysis.

## Results

The main characteristics of the samples are summarized in Table 2.

**Table 2:** Characteristics of RME and MME sample groups

		Male	Female	Mean age ± SD (year/month)		CVM stage	
				T0	T1	T0	T1
RME group	21	8	13	8.8 ± 1.37	9.6 ± 1.45	CS1–CS2	CS2–CS3
MME group	21	9	12	8.9 ± 2.39	10.5 ± 2.08	CS1–CS2	CS2–CS3

RME and MME groups were matched for number, sex, chronological age and CVMS. The RME group (*n* = 21) consisted of 13 girls and 8 boys with a mean age of 8.8 years ± 1.37 at T0 and 9.6 years ± 1.45 at T1. The MME group (*n* = 21) consisted of 12 girls and 9 boys with a mean age of 8.9 years ± 2.34 at T0 and 10.5 years ± 2.08 at T1. The CVMS ranged from 1 to 2 at T0 and from 2 to 3 at T1. The mean value of the method error was 0.5 ± 0.2 mm, within acceptable limits for the analysis of serial dental casts.

Descriptive statistics for values and changes of the dental measurements with comparisons are reported in Table 3.

**Table 3:** Dental cast analysis in RME and MME groups

	Initial					Change after treatment				Final				
	RME group		MME group			RME group		MME group		RME group		MME group		
	T0		T0			T1–T0		T1–T0		T1		T1		
Cast measure (mm)	Mean	SD	Mean	SD	*p* value	Mean	*p* value	Mean	*p* value	Mean	SD	Mean	SD	*p* value
Maxillary arch width														
Intermolar	43.2	1.576	41.62	3.72	ns	8.8	***	8.7	***	52	2.40	50.31	5.30	ns
Inter second premolar	38.39	1.784	36.93	2.78	ns	7.28	***	6.9	***	45.67	2.15	43.83	4.52	ns
Inter first premolar	33.9	1.698	32.66	2.23	ns	6.82	***	7.33	***	40.72	2.47	40	4.40	ns
Intercanine	29.32	2.135	28.62	2.26	ns	4.3	***	3.70	***	33.62	1.55	32.33	2.12	*
Mandibular arch width														
Intermolar	41.37	2.139	41.2	2.57	ns	1.5	*	2.09	**	42.87	2.35	43.29	2.62	ns
Inter second premolar	36.02	1.874	35.27	2.61	ns	1.17	*	2.36	**	37.2	1.69	37.63	2.79	ns
Inter first premolar	30.55	2.356	29.88	1.85	ns	0.8	ns	1.91	**	31.35	1.38	31.79	2.44	ns
Intercanine	24.9	1.382	24.45	1.50	ns	0.85	ns	1.13	*	25.75	1.58	25.59	1.68	ns
Maxillary arch depth	28.61	1.909	27.5	1.98	ns	−0.59	ns	−0.16	ns	28.02	2.80	27.34	2.18	ns
Mandibular arch depth	25.17	1.599	23.59	1.86	ns	0.3	ns	−0.59	ns	25.47	1.59	23	2.37	***

*ns* not significant

*p < 0.05; **p < 0.01; ***p < 0.001;

1. *Comparison of starting forms: RME group vs. MME group at T0*
  Before expansion treatment (T0), the groups were similar for all width and depth measurements for maxillary and mandibular arch examined (*p* > 0.05).
2. *Treatment effects: RME group T1–T0*
  In the RME group, significant increments in all the variables for maxillary and mandibular arch widths were observed after treatment. Maxillary intermolar, inter second premolar, inter first premolar and intercanine widths changed considerably (*p* < 0.001), while increases in mandibular intermolar and inter second premolar were slightly greater (*p* < 0.05). No significant differences in maxillary and mandibular arch depths were observed at the end of treatment.
3. *Treatment effects: MME group T1–T0*
  In the MME group, significant increases in all the variables for maxillary and mandibular arch widths were observed after treatment. Maxillary intermolar, inter second premolar, inter first premolar and intercanine widths changed considerably (*p* < 0.001), while increases in mandibular intermolar, inter second premolar, inter first premolar (*p* < 0.01) and intercanine widths (*p* < 0.05) were slightly greater. No significant differences in maxillary and mandibular arch depths were observed at the end of treatment.
4. *Comparison of final forms: RME group vs. MME group at T1*
  An evaluation of the changes after RME and MME (T1) showed statistically significant differences in mandibular arch depth (*p* < 0.001) and maxillary intercanine widths (*p* < 0.05). Differences in maxillary arch depth and arch width measurements were not significant.

## Discussion

The purpose of this retrospective study was to compare the changes in arch widths and depths on dental casts in two groups of pre-pubertal patients treated with MME or RME.

The method error was low, indicating the high reliability of procedures and measurements.

At baseline (T0), all the parameters were similar in the RME and MME groups (*p* > 0.05) and were thus comparable. All the upper measurements confirmed constriction of the dental arch associated with unilateral or bilateral posterior crossbite. In contrast, the lower values showed no abnormalities in arch widths, as reported in our previous study comparing the MME sample with an untreated control group [11].

After treatment, significant increases in upper and lower arch widths were observed in both the RME and MME group.

Changes in the upper arch intermolar, inter second premolar, inter first premolar and intercanine widths were significant (*p* < 0.001) in both groups.

Lower arch changes were significant for intermolar and inter second premolar in both the MME (*p* < 0.01) and RME (*p* < 0.05) group, whereas increases in inter first premolar (*p* < 0.01) and intercanine widths (*p* < 0.05) were significant only in the MME group. Conversely, no significant differences in upper and lower arch depth between the groups were noted.

Therefore, both procedures were found to be useful for increasing upper arch widths and gaining space to resolve unilateral or bilateral posterior crossbite and/or mild-to-moderate upper crowding.

However, as previously reported, the expansion obtained with MME presents minor dental side effects [22–24]. We may thus speculate that, although comparable at T1 in both groups, the increase in transverse diameters in the upper arch following MME is due to skeletal basal expansion rather than dental tipping. Upper arch changes obtained with MME are therefore subject to less relapse and consequently more stable.

A larger increase in lower widths was found in the MME group. This could be explained by a greater “lip bumper effect” observed after MME [23, 32]. When the upper widths increased slowly during the second step of activation, the cheeks were distanced from the buccal surfaces of mandibular teeth, causing the lower teeth to upright. Uprighting was greater in the molar than in the canine area as a consequence of greater posterior upper arch changes.

At T1, the two groups overlapped for all measurement increases, except for upper intercanine width (*p* < 0.05) and mandibular arch depth (*p* < 0.001). The difference in upper intercanine width is likely a result of the greater anterior opening of the mid-palatal suture, a consequence of increased skeletal expansion obtained by MME, as shown in our previous studies [22–24].

Interestingly, a statistically significant difference in mandibular arch depth between the two groups was observed. The loss in mandibular depth in the MME group might be related to the greater increase in transversal lower arch dimensions compared to the RME group. This study had some limitations. The first is due to the observational design although randomized controlled trials (RCTs) in orthodontic research are still very rare. Second, the small size of the sample can be explained with the very restrictive inclusion criteria. Third, the error method was low, but it does not mean high reliability, so the validity may be not high.

Our future goals are to compare the results obtained with MME and RME through the analysis of the cone beam for skeletal pattern and 3D model analysis for dental arches changes.

## Conclusions

RME and MME can be considered two effective treatment options to improve transverse arch dimensions and gain space in the dental arches. Few statistically significant differences in measurements of dental changes were found. RME and MME were both effective in increasing dental transverse dimensions by opening the mid-palatal suture in growing patients. A greater lower arch expansion was observed in the MME group, which might be attributed to the “lip bumper effects” observed in the MME protocol.
